# Supplementary material for: A glutamine-based single α-helix scaffold to target globular proteins
Source: Nat Commun. 2022 Nov 18;13:7073. doi: 10.1038/s41467-022-34793-6 (PMC9674830; doi:10.1038/s41467-022-34793-6)
Supplement: Supplementary file 1 — Supplementary Information [file 41467_2022_34793_MOESM1_ESM.pdf]

A glutamine-based single α-helix scaffold to target globular proteins

Supplementary Information

Albert Escobedo*[,1,9], Jonathan Piccirillo[1,10], Juan Aranda[1], Tammo Diercks[2], Borja Mateos[1], Carla Garcia-Cabau[1], Macarena Sánchez-Navarro[3], BusraTopal[1], Mateusz Biesaga[1], Lasse Staby[4], Birthe B. Kragelund[4], Jesús García[1], Oscar Millet[2], Modesto Orozco[1,5], Murray Coles[6], Ramon Crehuet[7] and Xavier Salvatella*[,1,8]

[1]Institute for Research in Biomedicine (IRB Barcelona), The Barcelona Institute of Science and Technology, Baldiri Reixac 10, 08028 Barcelona, Spain.

[2]CIC bioGUNE, Basque Research and Technology Alliance (BRTA), Bizkaia Science and Technology Park, 48160 Derio, Spain.

[3]Department of Molecular Biology, Instituto de Parasitología y Biomedicina López Neyra (IPBLN-CSIC), Armilla, Granada, Spain.

[4]REPIN and Structural Biology and NMR Laboratory, The Linderstrøm-Lang Centre for Protein Science, Department of Biology, Ole Maaloes Vej 5, University of Copenhagen, DK-2200 Copenhagen N, Denmark.

[5]Department of Biochemistry and Biomedicine, University of Barcelona, Avinguda Diagonal 645, 08028 Barcelona, Spain.

[6]Department of Protein Evolution, Max Planck Institute for Biology, Max-Planck-Ring 5, 72076 Tubingen, Germany.

[7]Institute for Advanced Chemistry of Catalonia (IQAC), CSIC, Jordi Girona 18-26, 08034 Barcelona, Spain.

[8]ICREA, Passeig Lluís Companys 23, 08010, Barcelona, Spain

*to whom correspondence should be addressed: albert.escobedo@crg.eu, xavier.salvatella@irbbarcelona.org

Current address:

[9]A. Escobedo, Centre for Genomic Regulation (CRG), The Barcelona Institute of Science and Technology, Barcelona, Spain.

[10]J. Piccirillo, Department of Macromolecular Structures, Centro Nacional de Biotecnología (CNB-CSIC), Madrid, Spain.

Supplementary Figures

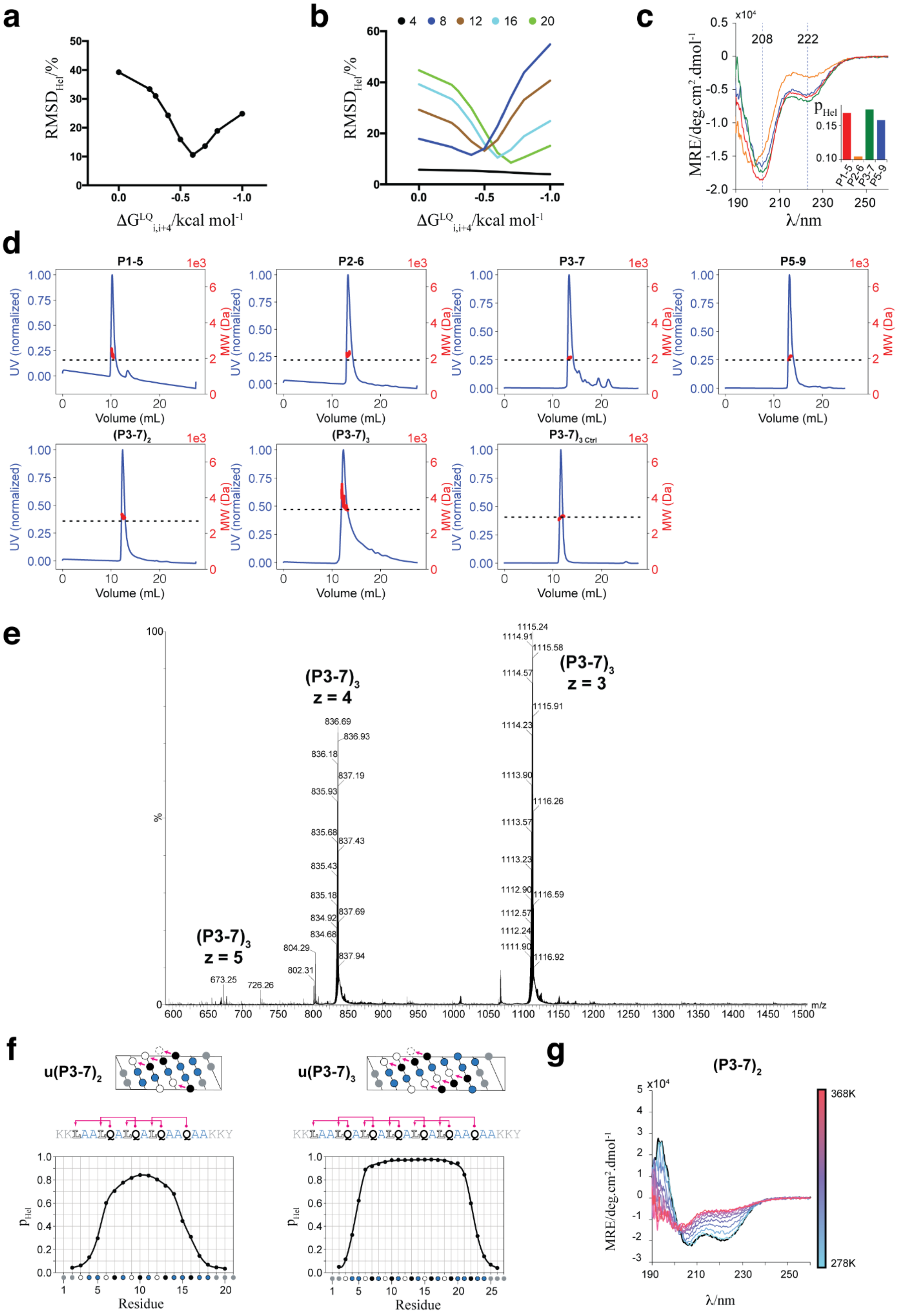

Supplementary Figure 1 | Cooperativity of Gln_{i+4}→Leu_i bifurcated hydrogen bonds in monomeric polyQ tracts and designed sequences. a Optimizing the value of a newly introduced energy term $\Delta G^{LQ}_{i,i+4}$ in *Agadir* to account for the Gln_{i+4}→Leu_i interaction improves its accuracy at predicting the helicity of L_4Q_{16} (Supplementary Table 2). **b** The optimal value of $\Delta G^{LQ}_{i,i+4}$ depends on polyQ tract length. Numbers in the legend indicate the length of the L_4Q_n peptides (full sequence $KKPGASL_4Q_nKKY$). **c** Left: overlay of the CD spectra obtained for the peptides in Fig. 1a. The dashed vertical lines highlight the positions where minima are observed in the CD spectrum of the α-helix (208 and 222 nm). In the inset, average peptide helicities are shown as obtained from deconvoluting the CD curve using the algorithm CONTIN (reference set 7) hosted at DichroWeb (http://dichroweb.cryst.bbk.ac.uk). **d** SEC-MALS analyses of the peptides in Fig. 1a and 1c. The horizontal dashed line indicates the molecular weight of the monomeric species calculated using the Protparam algorithm hosted at Expasy (https://web.expasy.org/protparam/). **e** Native mass spectrum of a ^{13}C-^{15}N labeled sample of the peptide $(P3\text{-}7)_3$, as obtained by Q-TOF MS using a Synapt G1-HDMS (see supplementary methods). Three ionization states corresponding to the monomeric species were independently detected. No oligomeric species were detected. **f** Chemical shifts-derived helical propensity (p_{Hel}) profiles of the $u(P3\text{-}7)_2$ and $u(P3\text{-}7)_3$ peptides, which are versions of the peptides shown in Fig. 1b lacking the PGAS N-capping sequence (the '*u*' stands for *uncapped*). **g** Thermal unfolding of $(P3\text{-}7)_2$. Shown are CD spectra acquired every 10 K in the 278 K - 368 K range (blue to red color gradient). The CD spectrum back at 278 K after thermal unfolding is shown in black.

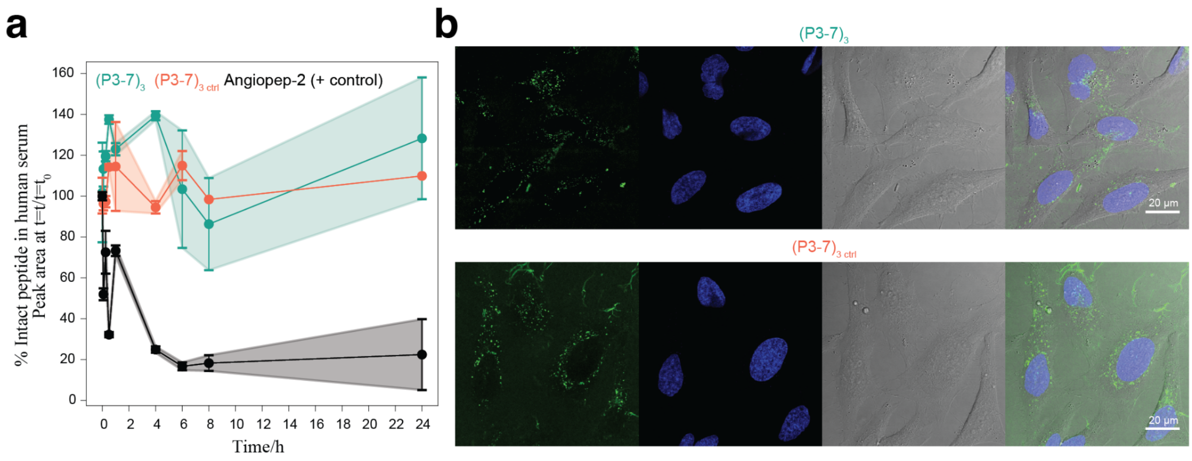

Supplementary Figure 2 | Stability in human serum and internalization in human cells of peptides (P3-7)$_3$ and (P3-7)$_{3\ Ctrl}$. **a** Human serum stability assay of peptides (P3-7)$_3$, (P3-7)$_{3\ Ctrl}$ and Angiopep-2 (sequence: TFFYGGSRGKRNNFKTEEY, a positive control for this assay [1]), measured at increasing time points by monitoring the area of the HPLC peak corresponding to the intact peptide over the area of the same peak at t = 0. Shown are mean values and the SD (error bars and shade) of three independent replicates. **b** Confocal microscopy images showing cytoplasmic internalization of peptides (P3-7)$_3$ (top) and (P3-7)$_{3\ Ctrl}$ (bottom) in HeLa cells. Cells were incubated with 90 µM solutions of AF488-labelled peptides for 90 minutes. The data are representative of three independent biological replicates. Left: AF488 emission showing internalization of the peptides in the cytoplasm. Center left: Hoechst 33342 emission of nuclear stain. Center right: optical images of the corresponding areas. Right: merge of all three images.

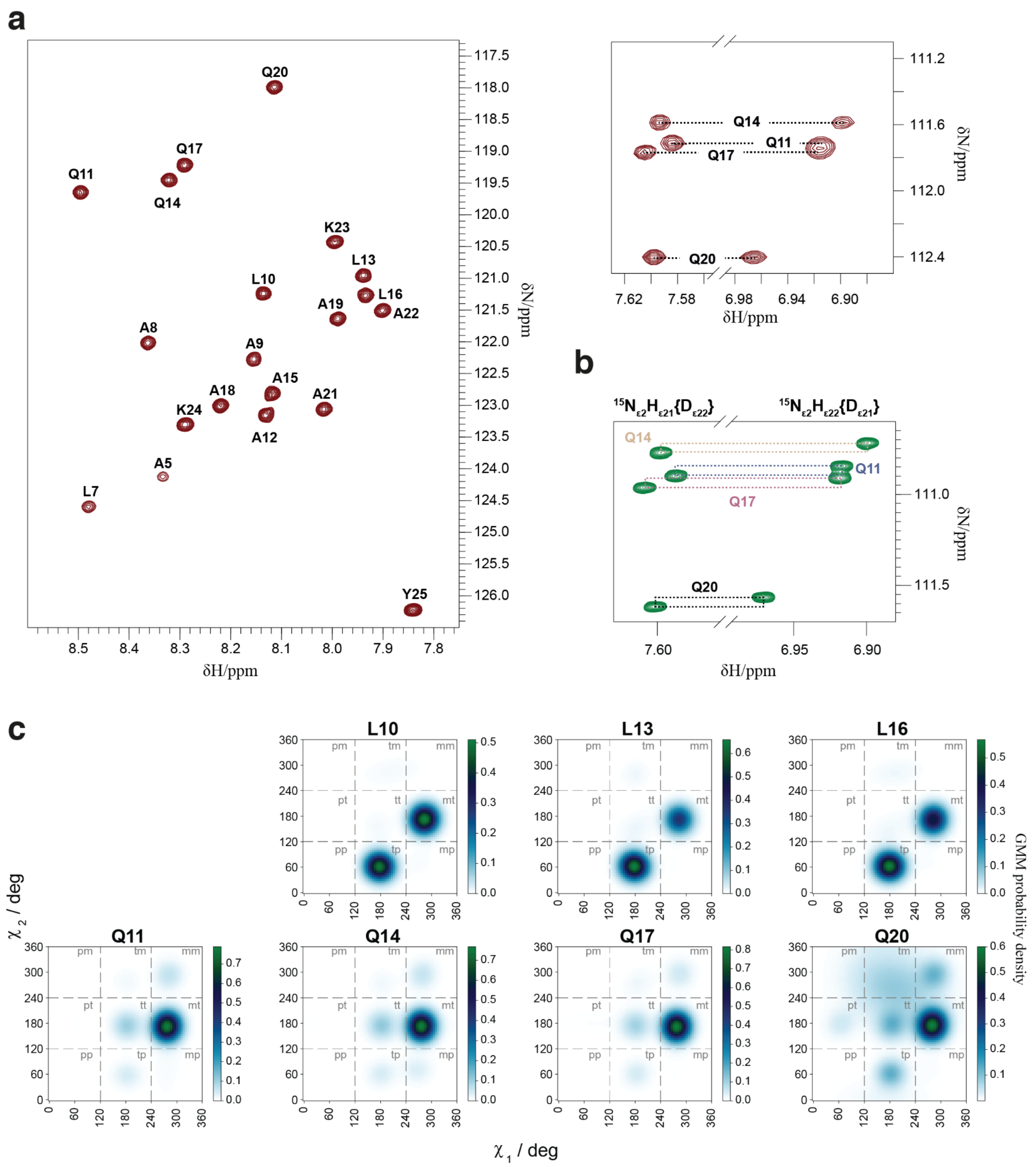

Supplementary Figure 3 | Structural features of $Gln_{i+4} \rightarrow Leu_i$ bifurcated hydrogen bonds: supporting analyses of the peptide $(P3\text{-}7)_2$. a Assigned ^{1}H-^{15}N HSQC spectrum of the peptide $(P3\text{-}7)_2$. Left: region of the spectrum displaying main chain N-H correlations. The spectrum shows wide signal dispersion in both dimensions and no signal overlap, which was exploited for the structural characterization shown in Fig. 2. Right: region of the spectrum showing the Q side chain NH_2 correlations. **b** Side chain region of a ^{1}H-^{15}N HSQC spectrum acquired on a $(P3\text{-}7)_2$ sample in 50% D_2O applying deuterium decoupling during the ^{15}N evolution time. The differential deuterium isotope shift in the NHD species results in different ^{15}N chemical shifts for each of them, which allows the unambiguous stereospecific assignment of the side chain carboxamide H_{ε} resonances[2] **c** Probability densities for χ_1 and χ_2 of all residues captured by the Gaussian Mixture Model (GMM) from the CoMAND per-residue fits.

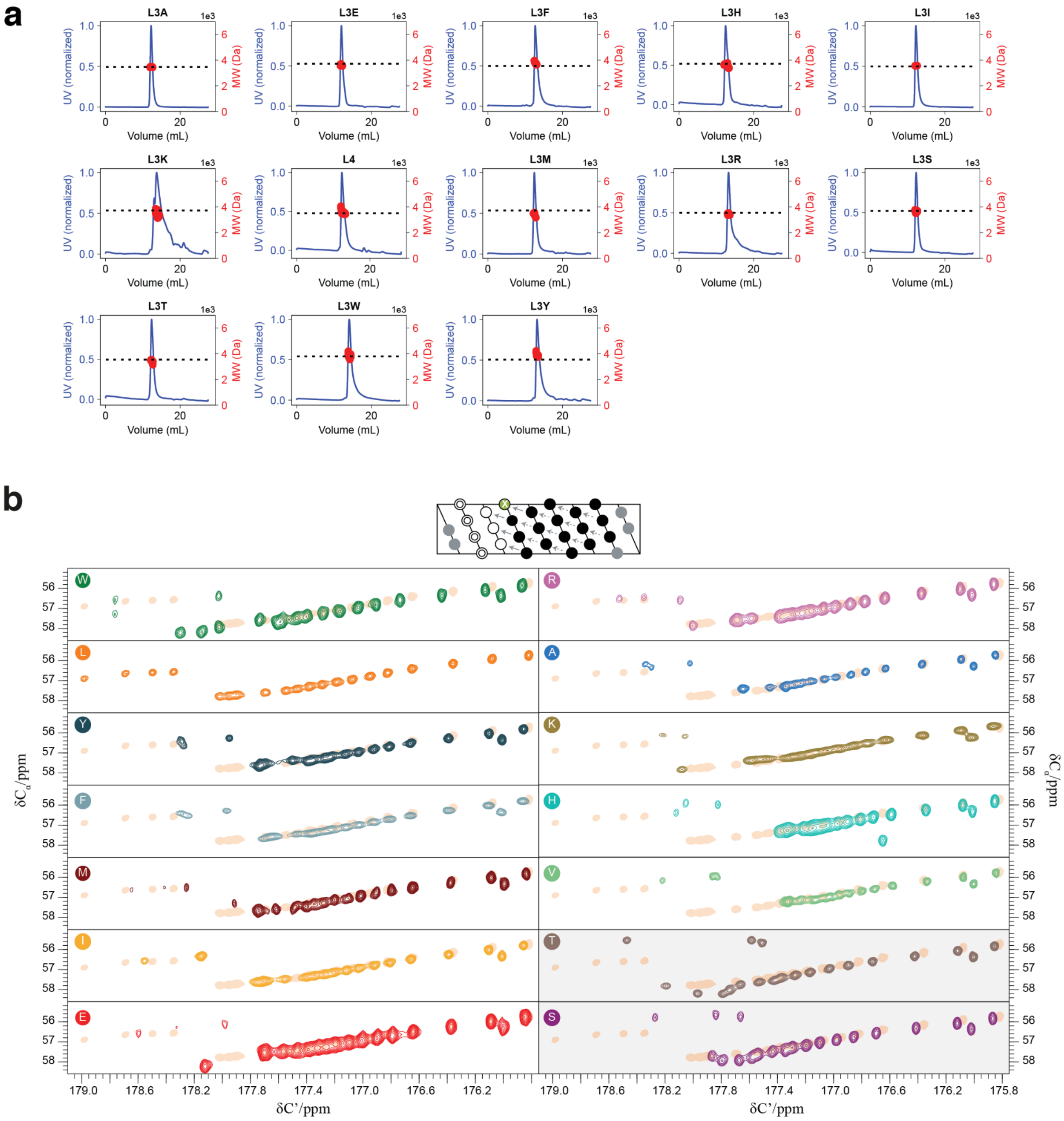

Supplementary Figure 4 | SEC-MALS analyses and CACO spectra of the L_3XQ_{16} peptides. a SEC-MALS analyses of the peptides in the L_3XQ_{16} series shown in Fig. 3. The horizontal dashed line indicates the molecular weight of the monomeric species calculated using the Protparam algorithm hosted at Expasy (https://web.expasy.org/protparam/). **b** ^{13}C-detected CACO spectra of the L_3XQ_{16} variants, sorted by their observed helicity, with the spectrum of L_4Q_{16} underlaid (orange shadow) in every panel for reference. Spectra shaded in gray indicate variants with outlier behavior.

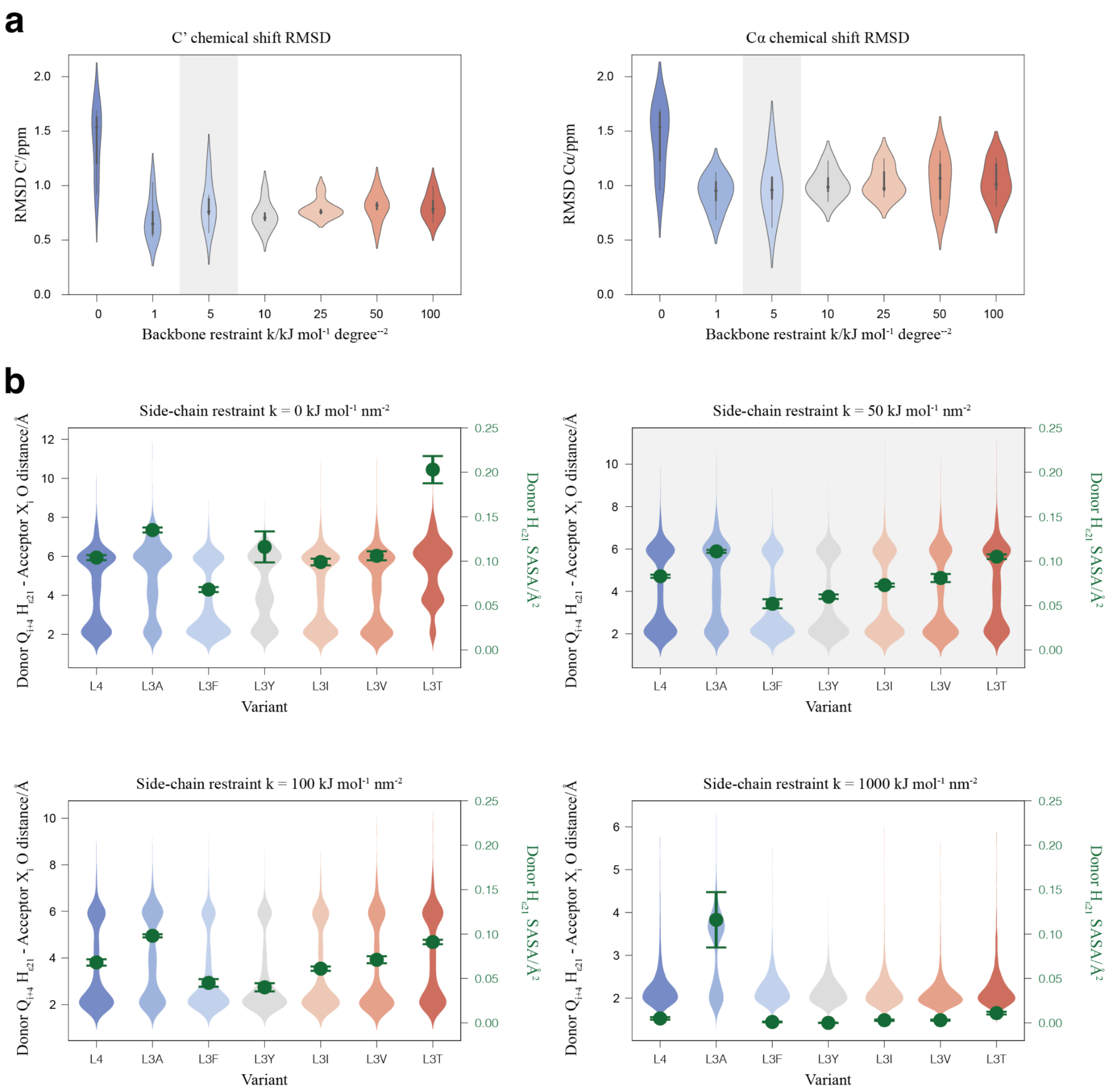

Supplementary Figure 5 | Parameter optimization for the MD simulations of the L_3XQ_{16} peptides with the Charmm36m force field. a Violin plots showing the distributions of C' and C_α RMSD values between measured and back-calculated chemical shifts, obtained in preparatory 500 ns trajectories of peptides L_4Q_{16}, L_3FQ_{16}, L_3YQ_{16}, L_3IQ_{16}, L_3VQ_{16} and L_3TQ_{16} (n = 6), with increasing strength of the spring constant (k) of a backbone restraint for the Φ (-60) and Ψ (-40) angles. In the inner boxplots, the center corresponds to the median, the boxplots boundaries correspond to the interquartile range (IQR) and the whiskers boundaries extend to 1.5 times the IQR. Chemical shift back-calculation was performed using the software PPM[3]. A value for k = 5 kJ mol^{-1} $degree^{-2}$ (shadowed area) was selected to minimize the RMSD and ensure a sensible sampling of the relevant conformations. **b** Mixed plots showing the distribution of distances between the donor Gln_{i+4} $H_{\varepsilon 21}$ and the acceptor X_i CO, as well as the SASA value of the donor Gln_{i+4} $H_{\varepsilon 21}$, obtained in preparatory 1 µs trajectories of peptides L_4Q_{16}, L_3AQ_{16}, L_3FQ_{16}, L_3YQ_{16}, L_3IQ_{16}, L_3VQ_{16} and L_3TQ_{16} (n = 4500 frames each), with a backbone restraint spring constant k = 5 kJ mol^{-1} $degree^{-2}$ and increasing strength of the spring constant (k) of a side chain restraint for the Gln_{i+4} $H_{\varepsilon 21}$ - X_i CO distance to be in the 0 - 4 Å range. SASA error bars represent the standard error of the mean, with the standard deviation obtained from dividing the 1 µs trajectories in 100 ns sub-blocks. A value of k = 50 kJ

mol^{-1} nm^{-2} (shadowed plot) was selected such that relevant conformations were sampled evenly across all trajectories, allowing for sufficient sampling of the side chain to main chain hydrogen bond while respecting the transient nature of the interaction, minimizing error and yielding sufficient dynamic range in SASA estimates.

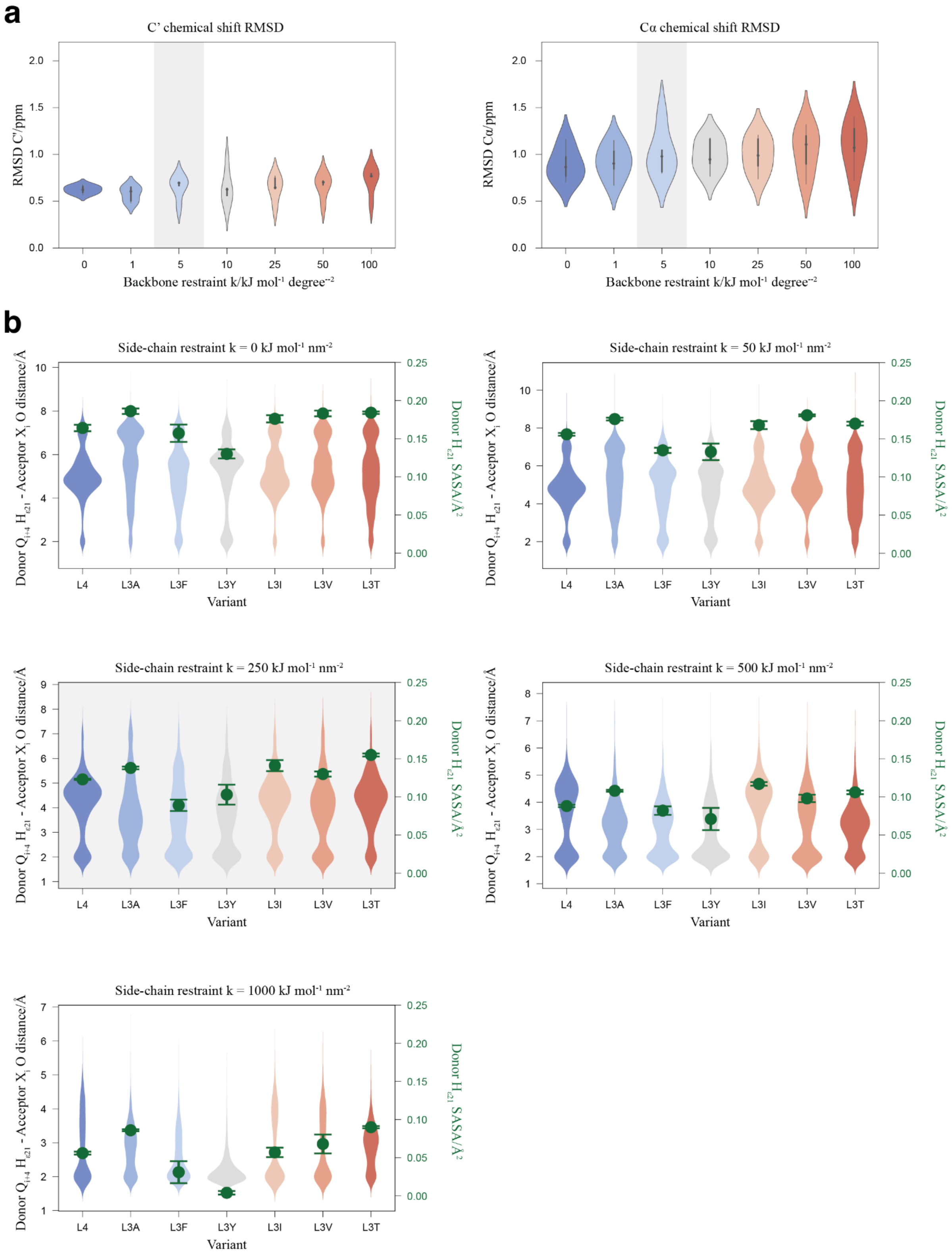

Supplementary Figure 6 | Parameter optimization for the MD simulations of the L_3XQ_{16} peptides with the a99sb force field. a Violin plots showing the distributions of C' and C_α RMSD values between measured and

back-calculated chemical shifts, obtained in preparatory 500 ns trajectories of peptides L_4Q_{16}, L_3FQ_{16}, L_3YQ_{16}, L_3IQ_{16}, L_3VQ_{16} and L_3TQ_{16} (n = 6), with increasing strength of the spring constant (k) of a backbone restraint for the Φ (-60) and Ψ (-40) angles. In the inner boxplots, the center corresponds to the median, the boxplots boundaries correspond to the interquartile range (IQR) and the whiskers boundaries extend to 1.5 times the IQR. Chemical shift back-calculation was performed using the software PPM[3]. A value for k = 5 kJ mol^{-1} $degree^{-2}$ (shadowed area) was selected to minimize the RMSD and ensure a sensible sampling of the relevant conformations. **b** Mixed plots showing the distribution of distances between the donor Gln_{i+4} $H_{\varepsilon 21}$ and the acceptor X_i CO, as well as the SASA value of the donor Gln_{i+4} $H_{\varepsilon 21}$, obtained in preparatory 1 μs trajectories of peptides L_4Q_{16}, $L_3AQ_{16,}$ L_3FQ_{16}, L_3YQ_{16}, L_3IQ_{16}, L_3VQ_{16} and L_3TQ_{16} (n = 4500 frames each), with a backbone restraint spring constant k = 5 kJ mol^{-1} $degree^{-2}$ and increasing strength of the spring constant (k) of a side chain restraint for the Gln_{i+4} $H_{\varepsilon 21}$ - X_i CO distance to be in the 0 - 4 Å range. SASA error bars represent the standard error of the mean, with the standard deviation obtained from dividing the 1 μs trajectories in 100 ns sub-blocks. A value of k = 250 kJ mol^{-1} nm^{-2} (shadowed plot) was selected such that relevant conformations were sampled evenly across all trajectories, allowing for sufficient sampling of the side chain to main chain hydrogen bond while respecting the transient nature of the interaction, minimizing error and yielding sufficient dynamic range in SASA estimates.

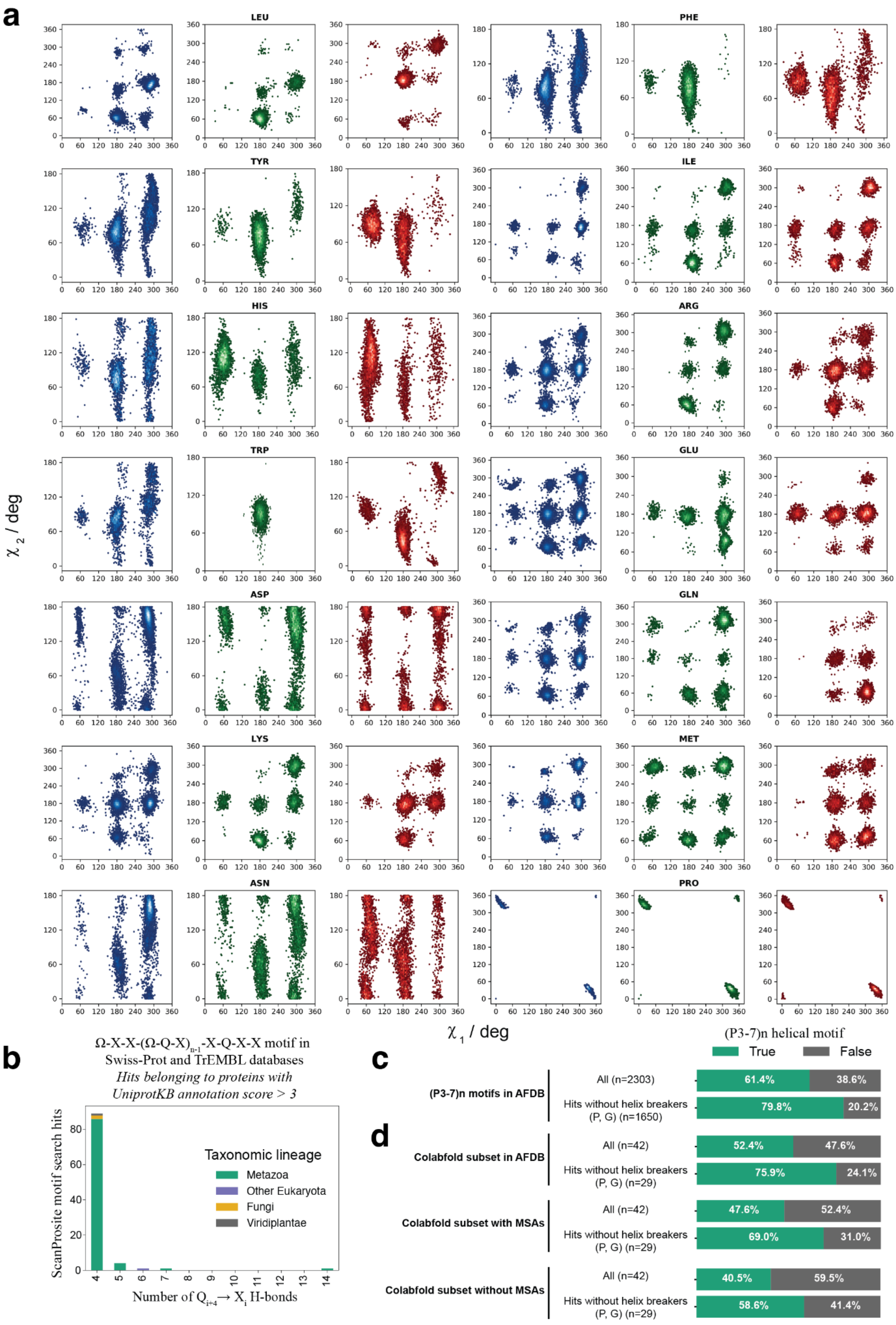
a
LEU
PHE
TYR
ILE
HIS
ARG
TRP
GLU
ASP
GLN
LYS
MET
ASN
PRO
χ 2 / deg
χ 1 / deg
b
Ω-X-X-(Ω-Q-X)n-1-X-Q-X-X motif in
Swiss-Prot and TrEMBL databases
Hits belonging to proteins with
UniprotKB annotation score > 3
ScanProsite motif search hits
Number of Qi+4 → Xi H-bonds
Taxonomic lineage
Metazoa
Other Eukaryota
Fungi
Viridiplantae
(P3-7)n helical motif
True
False
c
(P3-7)n motifs in AFDB
All (n=2303)
61.4%
38.6%
Hits without helix breakers (P, G) (n=1650)
79.8%
20.2%
d
Colabfold subset in AFDB
All (n=42)
52.4%
47.6%
Hits without helix breakers (P, G) (n=29)
75.9%
24.1%
Colabfold subset with MSAs
All (n=42)
47.6%
52.4%
Hits without helix breakers (P, G) (n=29)
69.0%
31.0%
Colabfold subset without MSAs
All (n=42)
40.5%
59.5%
Hits without helix breakers (P, G) (n=29)
58.6%
41.4%

Supplementary Figure 7 | Rotamer sampling in the L_3XQ_{16} MD simulations and supporting data on the natural sequences fulfilling our design rules. a Side chain χ_1-χ_2 dihedral angle correlations for residues with at least three side chain carbon atoms. In blue, values as reported in the BBRep database for residues located in the context of a helix. In green, values sampled throughout the Charmm36m 1 μs MD trajectories calculated for the corresponding L_3XQ_{16} variants. In red, values sampled throughout the a99sb-disp 1 μs MD trajectories calculated for the corresponding L_3XQ_{16} variants. **b** Number of ScanProsite-identified[4] protein sequences with an UniprotKB[5] annotation score > 3 found in the Swiss-Prot and TrEMBL databases containing the $(P3\text{-}7)_n$ motif (Ω = W, L, Y, F, I and M; X = any amino acid). Counts are grouped by taxonomic lineage as obtained from UniProt. **c** 2303 proteins containing sequence motifs fulfilling our design rules have models in the AlphaFold Database (AFDB) [6]. The top bar shows the percentage of these proteins where the $(P3\text{-}7)_n$-like motif is predicted to be helical from residue 1 to residue -4, as obtained from a DSSP analysis[7] of the structural models. The bottom bar shows the same for proteins not containing helix breaking residues (P, G) in the same segment (n=1650). **d** For a subset of proteins containing $(P3\text{-}7)_n$-like motifs, we predicted their structures with and without MSAs using ColabFold [8]. The top horizontal barplot shows the results of an analysis analogous to that shown in **c** for this subset of proteins in the AlphaFold DataBase. The central barplot shows the results of an analysis analogous to that shown in **c** for the same subset of proteins with their structure predicted with ColabFold using MSAs. The bottom barplot shows the results of an analysis analogous to that shown in **c** for the same subset of proteins with their structure predicted with ColaFold without the use of MSAs.

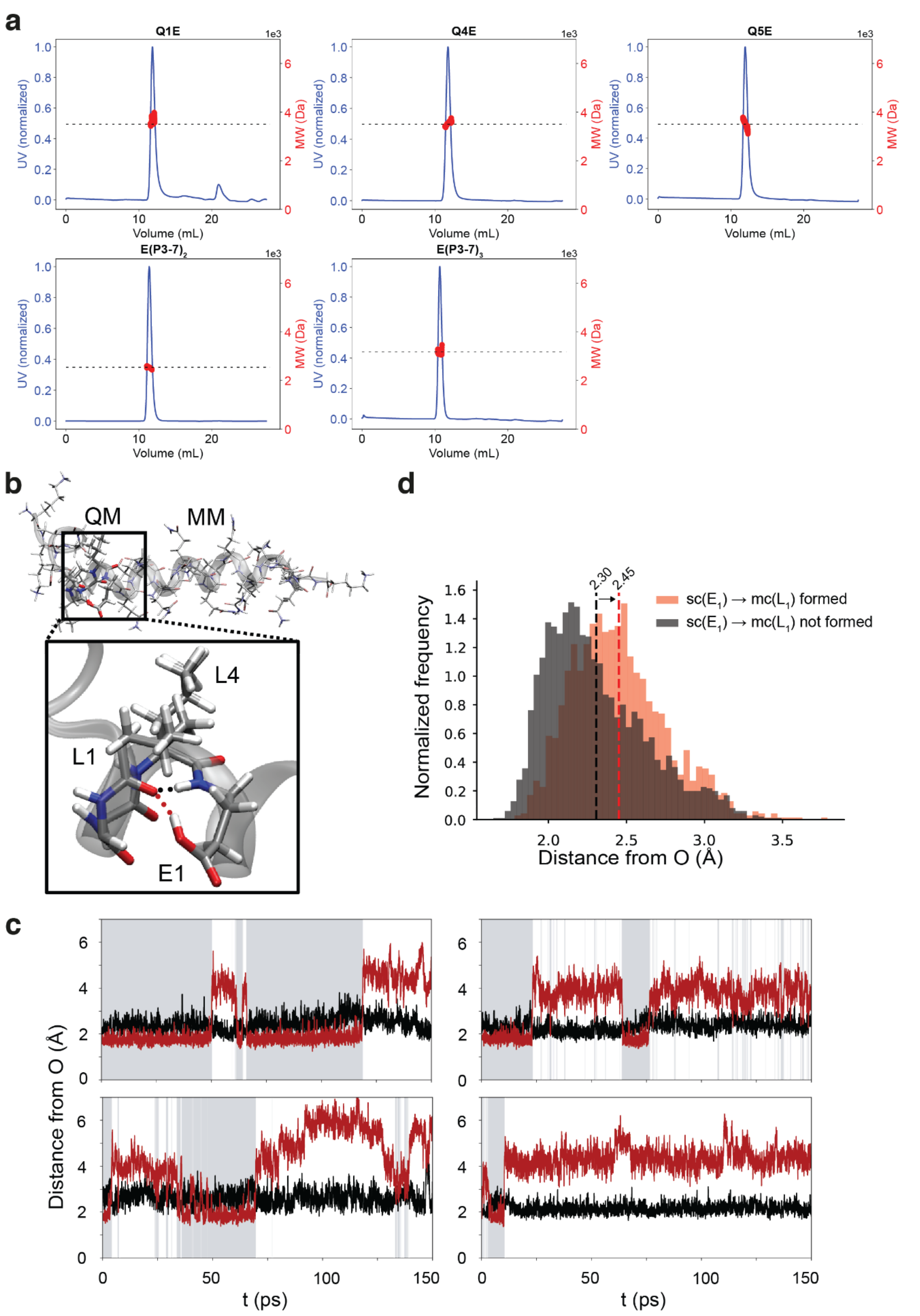

Supplementary Figure 8 | A pH-sensitive conformational switch based on glutamic acid bifurcated hydrogen bonds. a SEC-MALS analyses of the QXE and E(P3-7)$_n$ peptides shown in Fig. 4.The horizontal dashed line indicates the molecular weight of the monomeric species calculated using the Protparam algorithm

hosted at Expasy (https://web.expasy.org/protparam/). **b** Schematic representation of the QM/MM calculations setup. The atoms included in the QM subsystem are shown in sticks and the distances in **c** are indicated with dashed lines. **c** Time series of hydrogen bond donor-acceptor distances throughout four independent QM/MM trajectories. In black, H^N-O distance. In maroon, $H_{\varepsilon 2}$-O distance. Frames in which bifurcated hydrogen bonds are observed are indicated shadowed in gray. **d** Distribution of the distance between the main chain NH of E1 and the main chain CO of L1 in the absence and in the presence of the sc(E1)→mc(L1) hydrogen bond.

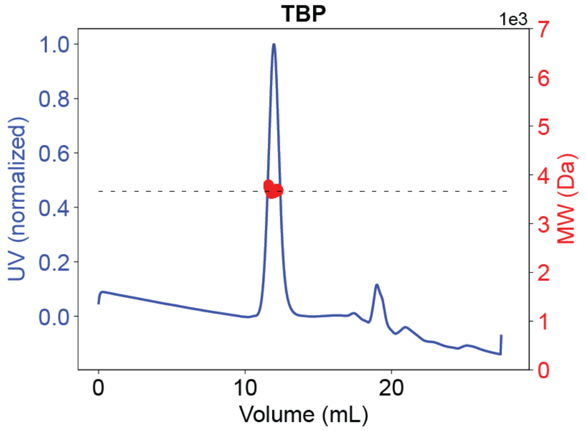

Supplementary Figure 9 | SEC-MALS analysis of the TBP polyQ peptide. Supporting SEC-MALS analysis for the peptide shown in Fig. 5. The horizontal dashed line indicates the molecular weight of the monomeric species calculated using the Protparam algorithm hosted at Expasy (https://web.expasy.org/protparam/).

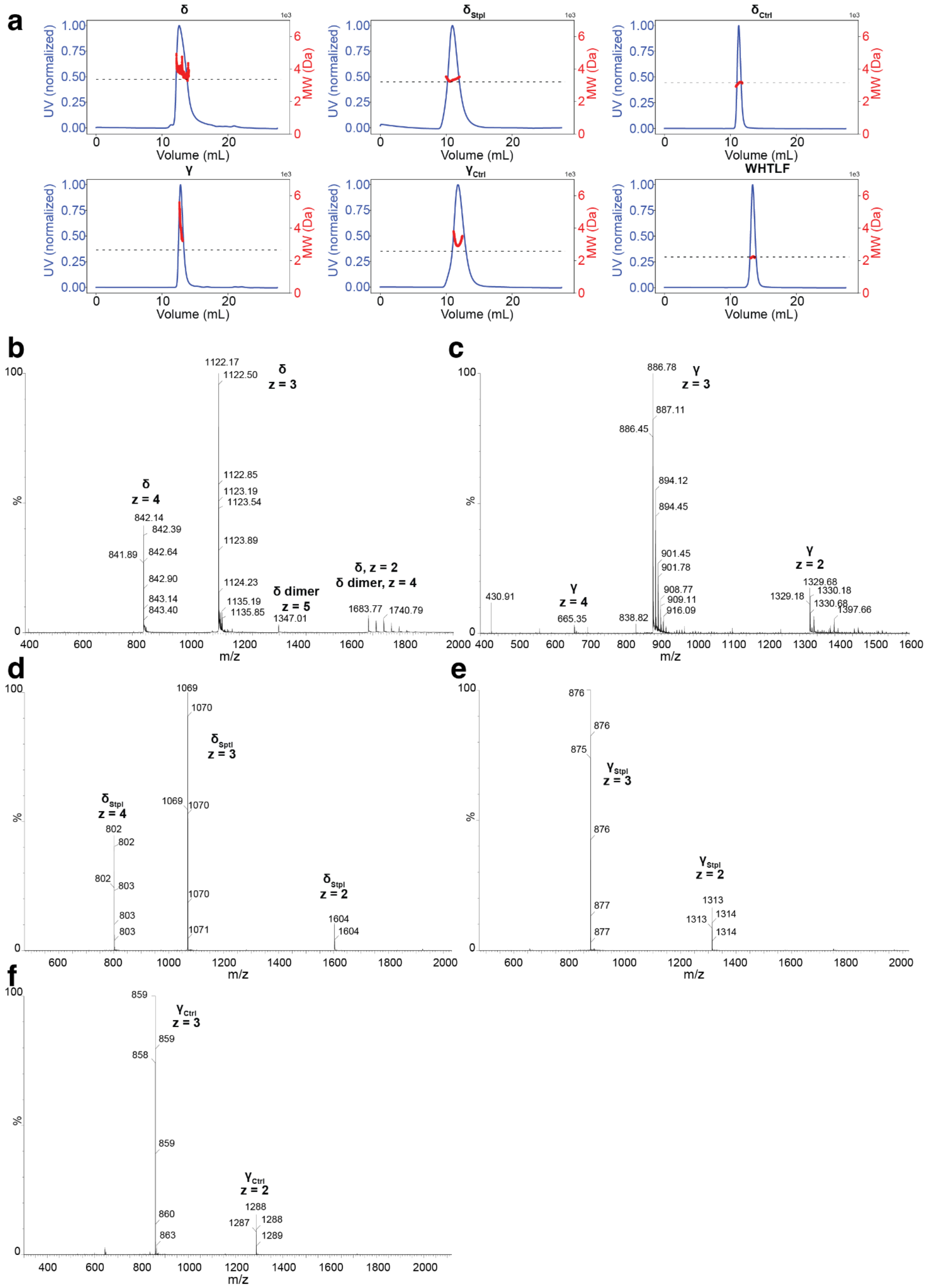

Supplementary Figure 10 | Design of binding motif-grafted peptides to bind the RAP74-CTD domain of TFIIF: supporting peptide analyses. a SEC-MALS analyses of the peptides shown in Fig. 6. The horizontal

dashed line indicates the molecular weight of the monomeric species calculated using the Protparam algorithm hosted at Expasy (https://web.expasy.org/protparam/). **b** Native mass spectrum of the peptide δ as obtained by Q-TOF MS using a Synapt G1-HDMS (see supplementary methods). Three ionization states corresponding to the monomeric species were independently detected. Two ionization states (one resolved, one ambiguous) corresponding to traces of the dimeric species were detected. **c** Native mass spectrum of the peptide γ. Three ionization states corresponding to the monomeric species were independently detected. No oligomeric species were detected. **d** Native mass spectrum of the peptide δ_{Stpl}. Three ionization states corresponding to the monomeric species were independently detected. No oligomeric species were detected. **e** Native mass spectrum of the peptide γ_{Stpl}. Two ionization states corresponding to the monomeric species were independently detected. No oligomeric species were detected. **f** Native mass spectrum of the peptide γ_{Ctrl}. Two ionization states corresponding to the monomeric species were independently detected. No oligomeric species were detected.

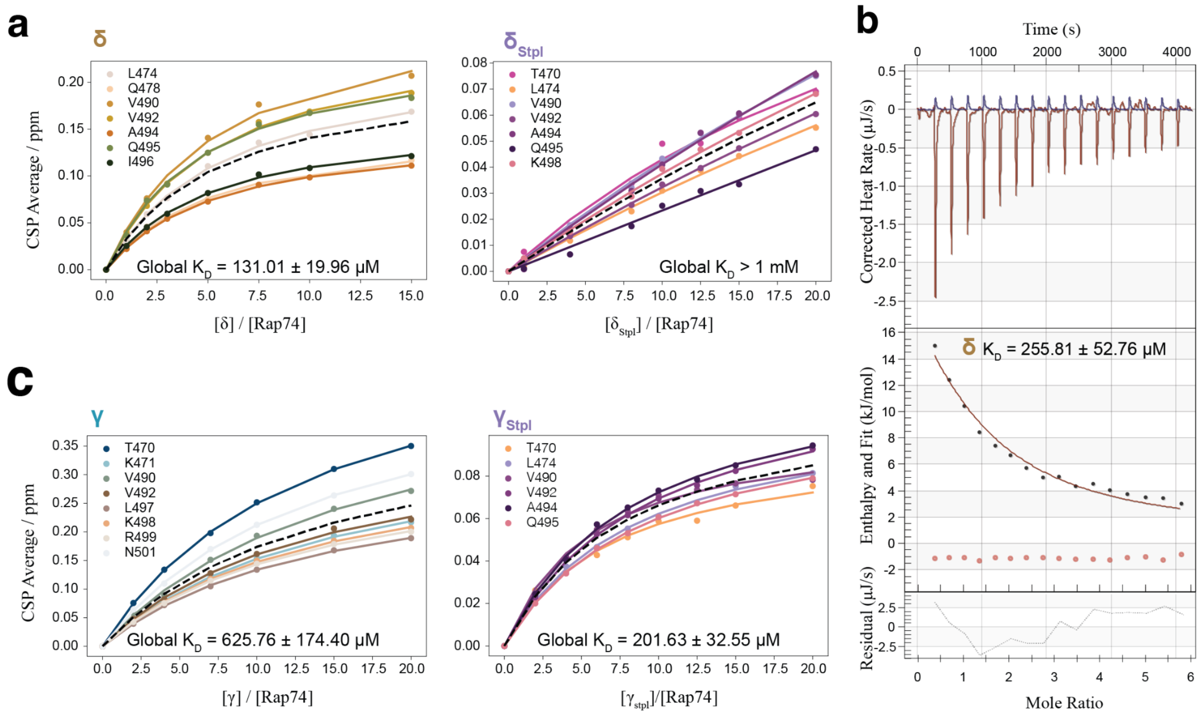

Supplementary Figure 11 | Design of binding motif-grafted peptides to bind the RAP74-CTD domain of TFIIF: supporting titration analyses. a K_D for the binding of the δ and the $δ_{Stpl}$ peptides to RAP74-CTD as obtained from a global fit (black dashed line) of the binding curves for the top 10% peaks with most intense averaged CSPs (individual fits shown as solid color lines). **b** ITC titration monitoring the binding of the δ peptide to RAP74-CTD. **c** As in **a** for the binding of the γ and the $γ_{Stpl}$ peptides to RAP74-CTD.

Supplementary Table 1 | CoMAND ensemble structure statistics. **a** R-factors averaged across the sequence (± SD) are given for the final ensemble compiled by global optimization (R_{mean}) and for ensembles compiled by residue-by-residue optimization (R_{opt}). **b** The coverage refers to the number of residues used in the analysis, versus the total number expected from the sequence. **c** Determined by MOLPROBITY [9]. The Ramachandran statistic lists the percentage of residues in favored / allowed / disfavored regions of the map (percentiles 98.0 / 99.8 / >99.8). Sidechain regularity lists the percentage in allowed sidechain rotamers (percentile 98.0). The clash score lists steric overlaps > 0.4 Å per 1000 atoms. **d** The RMSD to the average structure based on superimposition over ordered residues, as defined in the table.

**R-Factors[a]**	
R_{mean}	0.245 ± 0.075
R_{opt}	0.180 ± 0.027
Coverage[b]	15/22
**Covalent Geometry**	
Bonds (Å x 10^{-3})	29
Angles (°)	3.5
**Structure Quality Indicators[c]**	
Ramachandran Map (%)	98.1 / 0.9 / 0.0
Sidechain Regularity (%)	100
Clash Score	1.20
**Atomic R.M.S.D. (Å)[d]**	
Number of Structures	7
Ordered Residues	7-21
Backbone Heavy Atom	1.04 ± 0.57
All Heavy Atom	1.59 ± 0.56

Supplementary Table 2 | Details of the peptides used in this study. a No accompanying symbol indicates that the mass reported was calculated considering ^{12}C and ^{14}N isotopes using the Protparam algorithm hosted at Expasy (https://web.expasy.org/protparam/). † indicates the mass calculated for the ^{15}N enriched species and ‡ indicates the mass calculated for the ^{13}C-^{15}N enriched species. **b** Isotope labeling scheme as in **a**. In addition, no accompanying symbol indicates that the reported mass is an average of that measured during the corresponding peak elution using SEC-MALS, whereas * indicates that the reported mass is a z = 1 deconvoluted average of the main peaks corresponding to all detected ionization states in the native MS spectra.

**Name**	**Sequence**	**Expected mass (kDa)[a]**	**Observed mass (kDa)[b]**	**BMRB entry**
Cooperativity series (Fig. 1)				
**P1-5**	KKPGASLLAAQQAAAAKKY	1.92	2.23	51592
**P2-6**	KKPGASLALAQAQAAAKKY	1.92	2.19	51593
**P3-7**	KKPGASLAALQAAQAAKKY	1.92	2.03	51594
**P5-9**	KKPGASLAAAQLAAAQKKY	1.92	2.06	51595
**$(P3\text{-}7)_2$**	KKPGASLAALQALQALQAAQAAKKY	2.69‡	2.92‡	51591
**$(P3\text{-}7)_3$**	KKPGASLAALQALQALQALQALQAAQAAKKY	3.35‡	3.35‡*	51597
**$(P3\text{-}7)_{3\ Ctrl}$**	KKPGASAAAAQAAQAAQAAQAAQAAQAAKKY	2.91	2.92	N/A
L_3X series (Fig. 3)				
**L_3WQ_{16}**	KKPGASLLLWQQQQQQQQQQQQQQQQKKY	3.79‡	3.79‡	51578
**L_4Q_{16}**	KKPGASLLLLQQQQQQQQQQQQQQQQKKY	3.51	3.55	51616 and 27716
**L_3YQ_{16}**	KKPGASLLLYQQQQQQQQQQQQQQQQKKY	3.76‡	3.88‡	51579

**L_3FQ_{16}**	KKPGASLLLFQQQQQQQQQQQQQQQQKKY	3.74‡	3.76‡	51569
**L_3MQ_{16}**	KKPGASLLLMQQQQQQQQQQQQQQQQKKY	3.73‡	3.40‡	51573
**L_3IQ_{16}**	KKPGASLLLIQQQQQQQQQQQQQQQQKKY	3.51	3.56	51571
**L_3EQ_{16}**	KKPGASLLLEQQQQQQQQQQQQQQQQKKY	3.72‡	3.61‡	51568
**L_3RQ_{16}**	KKPGASLLLRQQQQQQQQQQQQQQQQKKY	3.55	3.42	51574
**L_3AQ_{16}**	KKPGASLLLAQQQQQQQQQQQQQQQQKKY	3.47	3.47	51567
**L_3KQ_{16}**	KKPGASLLLKQQQQQQQQQQQQQQQQKKY	3.52	3.45	51572
**L_3HQ_{16}**	KKPGASLLLHQQQQQQQQQQQQQQQQKKY	3.73‡	3.60‡	51570
**L_3VQ_{16}**	KKPGASLLLVQQQQQQQQQQQQQQQQKKY	3.49	N/A	51577
**L_3TQ_{16}**	KKPGASLLLTQQQQQQQQQQQQQQQQKKY	3.55†	3.40†	51576
**L_3SQ_{16}**	KKPGASLLLSQQQQQQQQQQQQQQQQKKY	3.68‡	3.60‡	51575
QXE series (Fig. 4)				
**Q1E**	KKPGASLLLLEQQQQQQQQQQQQQQQKKY	3.51	3.62	51580
**Q4E**	KKPGASLLLLQQQEQQQQQQQQQQQQKKY	3.51	3.53	51581
**Q5E**	KKPGASLLLLQQQQEQQQQQQQQQQQKKY	3.51	3.50	51582
**$E(P3\text{-}7)_2$**	KKPGASLAALEALEALEAAEAAKKY	2.54	2.53	N/A
**$E(P3\text{-}7)_3$**	KKPGASLAALEALEALEALEALEAAEAAKKY	3.17	3.12	51596

TBP (Fig. 5)				
**TBP**	KKNSLSILEEQQRQQQQQQQQQQQQQKKY	3.66	3.67	51583
Grafting series (Fig. 6)				
**δ**	KKPGASLAALQELQLLQLLQELQAAQAAKKY	3.36	3.36*	N/A
**δ_{Stpl}**	KKPGASAAAAQE**X**QLA**X**LAQELQAAQAAKKY	3.21	3.21*	N/A
**δ_{ctrl}**	KKPGASAAAAQEAQLAQLAQELQAAQAAKKY	3.15	3.13	N/A
**WHTLF**	SAAASSSWHTLFTAEEGQLYG	2.21	2.21	N/A
**γ**	KKPGASLSWLQLFQEEQAAQAAKK	2.66	2.66*	N/A
**γ_{Stpl}**	KKPGASASW**X**QLF**X**EEQAAQAAKK	2.62	2.62*	N/A
**γ_{Ctrl}**	KKPGASASWAQLFQEEQAAQAAKK	2.57	2.57*	N/A

Supplementary Table 3 | Sequences of the oligonucleotides used in this study. a 'S' stands for sumo, 'MBP' stands for maltose binding protein. **b** Synthetic genes were purchased from GeneArt (Thermo Fisher Scientific, Waltham, MA, USA) directly cloned into the expression pDEST-17 vector. PCR oligos were purchased as dry lyophilized material (Sigma-Aldrich, Burlington, MA, USA) and used as reagents in the Q5 Site-Directed Mutagenesis Kit from New England Biolabs (Ipswich, MA, USA) on the templates indicated in **c**.

Name[a]	Type[b]	Template[c]	Sequence
Cooperativity series (Fig. 1)			
**S-(P3-7)$_3$**	Synthetic gene	N/A	ATGTCGTACTACCATCACCATCACCATCACCTCGAATCAACAAGTTTGTACAAAAAAGCAGGCTTCATGAGCGATAGCGAAGTTAATCAAGAAGCCAAACCGGAAGTTAAGCCGGAAGTGAAACCTGAAACACATATTAACCTGAAAGTGAGTGATGGCAGCAGCGAAATCTTCTTCAAAATCAAAAAAACCACACCGCTGCGTCGTCTGATGGAAGCATTTGCAAAACGTCAGGGTAAAGAAATGGATAGCCTGCGTTTTCTGTATGATGGTATTCGTATTCAGGCAGATCAGACACCGGAAGATCTGGATATGGAAGATAACGATATTATCGAAGCACATCGTGAGCAGATTGGTGGTAAAAAACCGGGTGCAAGCCTGGCAGCACTGCAGGCGCTGCAGGCATTACAGGCACTGCAAGCCCTGCAAGCAGCCCAGGCAGCCAAAAAAATACTAATAA
**(P3-7)$_2$ fwd**	Oligo	S-(P3-7)$_3$	CAAGCCCTGCAAGCAGCC
**(P3-7)$_2$ rev**	Oligo	S-(P3-7)$_3$	CAGCGCCTGCAGTGCTGC
**u(P3-7)$_n$ fwd**	Oligo	S-(P3-7)$_n$	CTGGCAGCACTGCAGGCG
**u(P3-7)$_n$ rev**	Oligo	S-(P3-7)$_n$	TTTTTTACCACCAATCTGCTCACGATGTG
L_3X series (Fig. 3)			
**S-L$_4$Q$_{16}$**	Synthetic gene	N/A	ATGTCGTACTACCATCACCATCACCATCACCTCGAATCAACAAGTTTGTACAAAAAAGCAGGCTTCATGAGCGATAGCGAAGTTAATCAAGAAGCCAAACCGGAAGTTAAGCCGGAAGTGAAACCTGAAACACATATTAACCTGAAAGTGAGTGATGGCAGCAGCGAAATCTTCTTCAAAATCAAAAAAACCACACCGCTGCGTCGTCTGATGGAAGCATTTGCAAAACGTCAGGGTAAAGAAATGGATAGCCTGCGTTTTCTGTATGATGGTATTCGTATTCAGGCAGATCAGACACCGGAAGATCTGGATATGGAAGATAACGATATTATCGAAGCACATCGTGAGCAGATTGGTGGTAAAAAACCGGGTGCAAGCCTGCTGCTGCTTCAGCAGCAACAACAGCAGCAACAGCAACAGCAACAGCAGCAACAACAGAAAAAGTATTAATAA
**L$_3$WQ$_{16}$ fwd**	Oligo	S-L$_4$Q$_{16}$	CTGTGGCAGCAGCAACAACAGCAG
**L$_3$YQ$_{16}$ fwd**	Oligo	S-L$_4$Q$_{16}$	CTGTATCAGCAGCAACAACAGCAG

**L_3FQ_{16} fwd**	Oligo	S-L_4Q_{16}	CTGTTTCAGCAGCAACAACAGCAG
**L_3MQ_{16} fwd**	Oligo	S-L_4Q_{16}	CTGATGCAGCAGCAACAACAGCAG
**L_3IQ_{16} fwd**	Oligo	S-L_4Q_{16}	CTGATTCAGCAGCAACAACAGCAG
**L_3EQ_{16} fwd**	Oligo	S-L_4Q_{16}	CTGGAACAGCAGCAACAACAGCAG
**L_3RQ_{16} fwd**	Oligo	S-L_4Q_{16}	CTGCGTCAGCAGCAACAACAGCAG
**L_3AQ_{16} fwd**	Oligo	S-L_4Q_{16}	CTGGCCCAGCAGCAACAACAGCAG
**L_3KQ_{16} fwd**	Oligo	S-L_4Q_{16}	CTGAAACAGCAGCAACAACAGCAG
**L_3HQ_{16} fwd**	Oligo	S-L_4Q_{16}	CTGCATCAGCAGCAACAACAGCAG
**L_3VQ_{16} fwd**	Oligo	S-L_4Q_{16}	CTGGTGCAGCAGCAACAACAGCAG
**L_3TQ_{16} fwd**	Oligo	S-L_4Q_{16}	CTGACCCAGCAGCAACAACAGCAG
**L_3SQ_{16} fwd**	Oligo	S-L_4Q_{16}	CTGTCTCAGCAGCAACAACAGCAG
**L_3XQ_{16} rev**	Oligo	S-L_4Q_{16}	CAGCAGGCTTGCACCCGGTTTTTA
QXE series (Fig. 4)			
**S-Q1E**	Synthetic gene	N/A	ATGTCGTACTACCATCACCATCACCATCACCTCG<br>AATCAACAAGTTTGTACAAAAAAGCAGGCTTCAT<br>GAGCGATAGCGAAGTTAATCAAGAAGCCAAACCG<br>GAAGTTAAGCCGGAAGTGAAACCTGAAACACATA<br>TTAACCTGAAAGTGAGTGATGGCAGCAGCGAAAT<br>CTTCTTCAAAATCAAAAAAACCACACCGCTGCGT<br>CGTCTGATGGAAGCATTTGCAAAACGTCAGGGTA<br>AAGAAATGGATAGCCTGCGTTTTCTGTATGATGG<br>TATTCGTATTCAGGCAGATCAGACACCGGAAGAT<br>CTGGATATGGAAGATAACGATATTATCGAAGCAC<br>ATCGTGAGCAGATTGGTGGTAAAAAACCGGGTGC<br>AAGCCTGCTGCTGCTGGAACAGCAGCAACAACAG<br>CAACAGCAACAACAACAGCAGCAACAGCAAAAGA<br>AGTATTGATAA
**S-Q4E**	Synthetic gene	N/A	ATGTCGTACTACCATCACCATCACCATCACCTCG<br>AATCAACAAGTTTGTACAAAAAAGCAGGCTTCAT<br>GAGCGATAGCGAAGTTAATCAAGAAGCCAAACCG<br>GAAGTTAAGCCGGAAGTGAAACCTGAAACACATA<br>TTAACCTGAAAGTGAGTGATGGCAGCAGCGAAAT<br>CTTCTTCAAAATCAAAAAAACCACACCGCTGCGT<br>CGTCTGATGGAAGCATTTGCAAAACGTCAGGGTA<br>AAGAAATGGATAGCCTGCGTTTTCTGTATGATGG<br>TATTCGTATTCAGGCAGATCAGACACCGGAAGAT<br>CTGGATATGGAAGATAACGATATTATCGAAGCAC<br>ATCGTGAGCAGATTGGTGGTAAAAAACCGGGTGC<br>AAGCCTGCTGTTACTGCAGCAGCAAGAACAGCAG<br>CAACAGCAACAACAGCAGCAGCAACAGCAGAAAA

			AATACTAATAA
**S-Q5E**	Synthetic gene	N/A	ATGTCGTACTACCATCACCATCACCATCACCTCG<br>AATCAACAAGTTTGTACAAAAAAGCAGGCTTCAT<br>GAGCGATAGCGAAGTTAATCAAGAAGCCAAACCG<br>GAAGTTAAGCCGGAAGTGAAACCTGAAACACATA<br>TTAACCTGAAAGTGAGTGATGGCAGCAGCGAAAT<br>CTTCTTCAAAATCAAAAAAACCACACCGCTGCGT<br>CGTCTGATGGAAGCATTTGCAAAACGTCAGGGTA<br>AAGAAATGGATAGCCTGCGTTTTCTGTATGATGG<br>TATTCGTATTCAGGCAGATCAGACACCGGAAGAT<br>CTGGATATGGAAGATAACGATATTATCGAAGCAC<br>ATCGTGAGCAGATTGGTGGTAAAAAACCGGGTGC<br>AAGCCTGCTGTTACTGCAGCAGCAGCAAGAACAG<br>CAGCAACAGCAACAACAACAACAGCAGCAAAAGA<br>AATATTGATAA
**S-E(P3-7)$_3$**	Synthetic gene	N/A	ATGTCGTACTACCATCACCATCACCATCACCTCG<br>AATCAACAAGTTTGTACAAAAAAGCAGGCTTCAT<br>GAGCGATAGCGAAGTTAATCAAGAAGCCAAACCG<br>GAAGTTAAGCCGGAAGTGAAACCTGAAACACATA<br>TTAACCTGAAAGTGAGTGATGGCAGCAGCGAAAT<br>CTTCTTCAAAATCAAAAAAACCACACCGCTGCGT<br>CGTCTGATGGAAGCATTTGCAAAACGTCAGGGTA<br>AAGAAATGGATAGCCTGCGTTTTCTGTATGATGG<br>TATTCGTATTCAGGCAGATCAGACACCGGAAGAT<br>CTGGATATGGAAGATAACGATATTATCGAAGCAC<br>ATCGTGAGCAGATTGGTGGTAAAAAACCGGGTGC<br>AAGCCTGGCAGCACTGGAAGCCCTGGAAGCGTTA<br>GAGGCTTTAGAGGCGCTGGAAGCAGCAGAAGCAG<br>CCAAAAAAATACTGATAA
TBP (Fig. 5)			
**S-TBP**	Synthetic gene	N/A	ATGTCGTACTACCATCACCATCACCATCACCTCG<br>AATCAACAAGTTTGTACAAAAAAGCAGGCTTCAT<br>GAGCGATAGCGAAGTTAATCAAGAAGCCAAACCG<br>GAAGTTAAGCCGGAAGTGAAACCTGAAACACATA<br>TTAACCTGAAAGTGAGTGATGGCAGCAGCGAAAT<br>CTTCTTCAAAATCAAAAAAACCACACCGCTGCGT<br>CGTCTGATGGAAGCATTTGCAAAACGTCAGGGTA<br>AAGAAATGGATAGCCTGCGTTTTCTGTATGATGG<br>TATTCGTATTCAGGCAGATCAGACACCGGAAGAT<br>CTGGATATGGAAGATAACGATATTATCGAAGCAC<br>ATCGTGAGCAGATTGGTGGCAAAAAAAACAGCCT<br>GAGCATTCTGGAAGAACAGCAGCGTCAGCAGCAA<br>CAACAGCAGCAACAGCAACAGCAGCAGCAGAAAA<br>AATACTAATAA
Grafting series (Fig. 6)			
**MBP-RAP74-CTD**	Synthetic gene	N/A	ATGAAAATCCATCACCATCACCATCACGAAGAAG<br>GTAAACTGGTAATCTGGATTAACGGCGATAAAGG

			CTATAACGGTCTCGCTGAAGTCGGTAAGAAATTC<br>GAGAAAGATACCGGAATTAAAGTCACCGTTGAGC<br>ATCCGGATAAACTGGAAGAGAAATTCCCACAGGT<br>TGCGGCAACTGGCGATGGCCCTGACATTATCTTC<br>TGGGCACACGACCGCTTTGGTGGCTACGCTCAAT<br>CTGGCCTGTTGGCTGAAATCACCCCGGACAAAGC<br>GTTCCAGGACAAGCTGTATCCGTTTACCTGGGAT<br>GCCGTACGTTACAACGGCAAGCTGATTGCTTACC<br>CGATCGCTGTTGAAGCGTTATCGCTGATTTATAA<br>CAAAGATCTGCTGCCGAACCCGCCAAAAACCTGG<br>GAAGAGATCCCGGCGCTGGATAAAGAACTGAAAG<br>CGAAAGGTAAGAGCGCGCTGATGTTCAACCTGCA<br>AGAACCGTACTTCACCTGGCCGCTGATTGCTGCT<br>GACGGGGGTTATGCGTTCAAGTATGAAAACGGCA<br>AGTACGACATTAAAGACGTGGGCGTGGATAACGC<br>TGGCGCGAAAGCGGGTCTGACCTTCCTGGTTGAC<br>CTGATTAAAAACAAACACATGAATGCAGACACCG<br>ATTACTCCATCGCAGAAGCTGCCTTTAATAAAGG<br>CGAAACAGCGATGACCATCAACGGCCCGTGGGCA<br>TGGTCCAACATCGACACCAGCAAAGTGAATTATG<br>GTGTAACGGTACTGCCGACCTTCAAGGGTCAACC<br>ATCCAAACCGTTCGTTGGCGTGCTGAGCGCAGGT<br>ATTAACGCCGCCAGTCCGAACAAAGAGCTGGCAA<br>AAGAGTTCCTCGAAAACTATCTGCTGACTGATGA<br>AGGTCTGGAAGCGGTTAATAAAGACAAACCGCTG<br>GGTGCCGTAGCGCTGAAGTCTTACGAGGAAGAGT<br>TGGCGAAAGATCCACGTATTGCCGCCACCATGGA<br>AAACGCCCAGAAAGGTGAAATCATGCCGAACATC<br>CCGCAGATGTCCGCTTTCTGGTATGCCGTGCGTA<br>CTGCGGTGATCAACGCCGCCAGCGGTCGTCAGAC<br>TGTCGATGAAGCCCTGAAAGACGCGCAGACTAAT<br>TCGATCACAAGTTTGTACAAAAAAGCAGGCTCCG<br>AAAACCTGTACTTCCAGGGCGACGTGCAGGTGAC<br>TGAGGATGCCGTGCGCCGCTACCTGACACGGAAG<br>CCCATGACCACTAAGGACCTGCTGAAAAAGTTCC<br>AGACCAAGAAGACAGGGCTGAGCAGCGAGCAGAC<br>AGTGAACGTGTTGGCCCAGATCCTCAAGCGACTC<br>AACCCCGAGCGCAAGATGATCAACGACAAAATGC<br>ACTTCTCTCTCAAGGAGTAA

Supplementary Table 4 | Residual Dipolar Couplings (RDCs) measured for (P3-7)$_2$. a RDCs were measured in isotopically-labeled (P3-7)$_2$ embedded in sterically aligning 7% acrylamide gels as detailed in Methods. Also available for download at BMRB entry 51591. **b** RDCs were back-calculated from each individual frame generated in all 20 ensemble calculation iterations, averaged and scaled to minimize the RMSD against experimental measurements as explained in Methods.

RDC Code	Residue Number	Residue Type	Atom 1	Atom 2	Measured RDC (Hz)[a]	Ensemble-derived RDC (Hz)[b]
^{1}DNH	4	GLY	N	H	2.463	13.986
^{1}DNH	5	ALA	N	H	-2.287	9.853
^{1}DNH	7	LEU	N	H	-11.448	-6.195
^{1}DNH	8	ALA	N	H	-17.047	-12.526
^{1}DNH	9	ALA	N	H	-18.799	-18.783
^{1}DNH	10	LEU	N	H	-15.850	-10.207
^{1}DNH	11	GLN	N	H	-17.068	-13.222
^{1}DNH	12	ALA	N	H	-20.043	-21.474
^{1}DNH	13	LEU	N	H	-18.776	-19.659
^{1}DNH	14	GLN	N	H	-16.706	-16.388
^{1}DNH	15	ALA	N	H	-18.258	-14.576
^{1}DNH	16	LEU	N	H	-19.302	-19.634
^{1}DNH	17	GLN	N	H	-16.437	-13.138
^{1}DNH	18	ALA	N	H	-14.323	-6.859
^{1}DNH	19	ALA	N	H	-15.520	-14.234
^{1}DNH	20	GLN	N	H	-12.660	-16.050
^{1}DNH	21	ALA	N	H	-7.799	-3.117
^{1}DNH	22	ALA	N	H	-6.211	-3.211
^{1}DNH	23	LYS	N	H	-0.653	-4.646
^{1}DNH	24	LYS	N	H	6.471	6.901
^{1}DNC	5	ALA	N	C	0.905	-0.512
^{1}DNC	7	LEU	N	C	0.680	-0.440
^{1}DNC	8	ALA	N	C	0.710	-0.215
^{1}DNC	9	ALA	N	C	-0.456	0.017
^{1}DNC	10	LEU	N	C	0.450	-0.221
^{1}DNC	11	GLN	N	C	1.262	-0.297
^{1}DNC	12	ALA	N	C	1.731	-0.104
^{1}DNC	13	LEU	N	C	0.610	0.122
^{1}DNC	14	GLN	N	C	0.754	-0.293
^{1}DNC	15	ALA	N	C	1.563	-0.226
^{1}DNC	16	LEU	N	C	2.178	0.010
^{1}DNC	17	GLN	N	C	1.234	-0.016
^{1}DNC	18	ALA	N	C	1.421	-0.279

**[1]DNC**	19	ALA	N	C	0.281	-0.140
**[1]DNC**	20	GLN	N	C	-0.584	0.049
**[1]DNC**	21	ALA	N	C	1.300	-0.041
**[1]DNC**	22	ALA	N	C	0.672	-0.108
**[1]DNC**	23	LYS	N	C	0.987	-0.080
**[1]DNC**	24	LYS	N	C	0.328	-0.059
**[1]DCH**	5	ALA	C	H	-0.540	-2.013
**[1]DCH**	7	LEU	C	H	-0.390	-1.905
**[1]DCH**	8	ALA	C	H	0.420	-1.328
**[1]DCH**	9	ALA	C	H	0.330	-0.554
**[1]DCH**	10	LEU	C	H	-1.302	-1.194
**[1]DCH**	11	GLN	C	H	-0.288	-1.856
**[1]DCH**	12	ALA	C	H	0.006	-0.919
**[1]DCH**	13	LEU	C	H	-0.444	-0.193
**[1]DCH**	14	GLN	C	H	1.470	-1.650
**[1]DCH**	15	ALA	C	H	-0.360	-1.599
**[1]DCH**	16	LEU	C	H	3.120	-0.537
**[1]DCH**	17	GLN	C	H	-0.372	-0.488
**[1]DCH**	18	ALA	C	H	-0.294	-1.455
**[1]DCH**	19	ALA	C	H	-0.174	-1.104
**[1]DCH**	20	GLN	C	H	-0.258	-0.026
**[1]DCH**	21	ALA	C	H	-0.546	-0.324
**[1]DCH**	22	ALA	C	H	-1.116	-1.127
**[1]DCH**	23	LYS	C	H	-1.116	-0.776
**[1]DCH**	24	LYS	C	H	-0.918	-0.030

Supplementary Methods

Parametrization of $Q_{(i+4)} \rightarrow L_{(i)}$ side chain to main chain hydrogen bonds in Agadir

Agadir source code was kindly shared by Professor Luis Serrano (Center for Genomic Regulation, Barcelona, Spain). The energy term accounting for Leu_i - Gln_{i+4} interactions $\Delta G^{LQ}_{i,i+4}$ was progressively increased in the 0 to 1 kcal mol^{-1} range. Chemical shift-derived (BMRB entries 27713, 27714, 27715, 27716, 27717) experimental per-residue helical propensity (p_{hel}) values for the L_4Q_n peptides (full sequence $KKPGASL_4Q_nKK$) were previously reported[10]. At each $\Delta G^{LQ}_{i,i+4}$ increase step, agadir was used to predict per-residue helicity and the RMSD between the predicted and experimentally determined per-residue helical content was obtained using:

$$RMSD_{Hel} = \sqrt{\frac{1}{peptide\ length} \sum_{i=1}^{peptide\ length} \left(Hel\%_i^{experimental} - Hel\%_i^{predicted}(E^L_{i,i+4})\right)^2}$$

SEC-MALS analyses

The oligomeric state of peptides in solution was determined by size exclusion chromatography coupled to multiple angle light scattering. Peptide samples were loaded in a Superdex Peptide 10/300 GL column (GE Healthcare, Chicago, IL, USA) mounted on a Shimadzu Prominence Modular HPLC with a SPD-20 UV detector (Shimadzu, Kyoto, Japan) coupled to a Dawn Heleos-II multi-angle light scattering detector (18 angles, 658 nm laser beam) and an Optilab T-rEX refractometer (Wyatt Technology, Santa Barbara, CA, USA). The SEC-UV/MALS/RI system was equilibrated with sodium phosphate 20 mM, 0.1% TFA at 298 K with a 0.5 mL min^{-1} flow rate and elution was monitored using UV absorbance at 280 nm over 55 minutes. Data acquisition and processing was performed using the Astra 6.1 software (Wyatt Technology, Santa Barbara, CA, USA).

Native Mass Spectrometry

Native mass spectrometry was used to determine the oligomeric state of some peptides. MS experiments were performed using a Synapt G1-HDMS mass spectrometer (Waters, Manchester, UK). All samples were buffer exchanged into 150 mM ammonium acetate and were infused by automated chip-based nanoelectrospray using a Triversa Nanomate system (Advion BioSciences, Ithaca, NY, USA) as the interface. The ionization was performed in positive mode using a spray voltage and a gas pressure of 1.75 kV and 0.5 psi, respectively. The source pumping speed in the backing region (5.85 mbar) of the mass spectrometer was

reduced to achieve optimal transmission of non-covalent complexes. Cone voltage, extraction cone and source temperature were set to 40 V, 6 V and 313 K, respectively. Trap and transfer collision energies were set to 6 V and 4 V, respectively. The pressure in the Trap and Transfer T-Wave regions were $2.39 \cdot 10^{-2}$ mbar of Ar and the pressure in the IMS T-Wave was 0.467 mbar of N_2. Trap gas and IMS gas flows were 8 and 25 mL/sec, respectively. The instrument was calibrated over the 300-8000 Da m/z range using a solution of cesium iodide. MassLynx version 4.1 SCN 704 was used for data processing.

Peptide stability in human serum

1.5 mM solutions of the $P(3\text{-}7)_3$, $P(3\text{-}7)_{3\,Ctrl}$ and Angiopep-2 peptides were prepared as detailed above in 20 mM sodium phosphate, pH 7.4 and diluted in human serum (Sigma-Aldrich, Burlington, MA, USA) (1:9 dilution) to a final concentration of 300 µM. The incubation was performed at 310 K for 24 h, taking aliquots of 50 µL at several time points. These samples were treated with 400 µL of methanol to precipitate the serum proteins and centrifuged for 30 min at 277 K. The supernatant was filtered and analyzed by HPLC to calculate the percentage of intact peptide in the sample. HPLC chromatograms were obtained on an Agilent 1260 Infinity II system with a PDA detector, with an Agilent Poroshell 120-EC18 (50 x 3 mm x 2.7 µm) column. The flow rate was 1 mL min^{-1} and acetonitrile (1% TFA) and water (1% TFA) were used as solvents, with 4 minute linear gradients from 5 % to 95 % of acetonitrile thermostated at 313 K.

Peptide internalization experiments in HeLa cells

1.5 equivalents of AF488-NHS (Thermofisher, ref. A20000, 10 mg mL^{-1}, DMSO) were added to solutions of $P(3\text{-}7)_3$ o $P(3\text{-}7)_{3\,Ctrl}$ in 20 mM sodium phosphate, pH 7.4, in three steps every 10 min and left to react for 30 min. The excess of dye was removed by SEC (PD MiniTrap G10, Cytiva, following manufacturer's instructions) and the peptide was stored at 277 K in PBS protected from light.

HeLa cells (ATCC, Manassas, VA) were cultured in DMEM complete medium (glucose 4.5 g L^{-1}, 2 mM glutamine, 10% FBS and 0.5% Pen/Strep) (Sigma-Aldrich, Burlington, MA, USA). Medium was changed 3 times per week and cells were detached using 0.05% trypsin/EDTA. The day before the experiment, 30000 cells/well were seeded on an 8-well chamber slide with a coverslip-like bottom (µ-Slide 8 Well from ibidi, cat. 80826) previously coated with collagen 1 h at room temperature. The cells were incubated at 310 K and 5% of CO_2. The day of the experiment, the cells were incubated with AF488 labeled peptides (90 µM) for up

to 120 minutes. After the experiment, the peptides were removed and the cells were washed with PBS prior visualization.

Images were acquired on a Leica TCS-SP8 confocal microscope using a 63x 1.4NA oil objective (HCX PLAN APO CS). Images were collected using single excitation for each wavelength separately. Hoechst 33342 was excited with the 405 nm UV diode and 430-466 nm emission band pass, and AF488 was excited with white laser (488 nm) and 509-537 nm emission bandpass.

Later 15-20 image sections of selected areas were acquired with a step size of 0.25 µm, and z-stack images analyzed and processed using the Fiji distribution of ImageJ.

Super-resolution images were acquired on the SP8 CONFOCAL MICROSCOPE lightning mode. The SP8 confocal microscope allows working in super-resolution around 120 nm. The same settings were used for image acquisition of selected areas.

Isothermal Titration Calorimetry (ITC)

A 1.5 mM solution of the δ peptide was titrated into 190 µL of a 75 µM RAP74-CTD solution (both in 20 mM sodium phosphate, pH 7.4, jointly equilibrated in a two-step dialysis) at 298 K using a NanoITC calorimeter (TA Instruments). The peptide was titrated with one initial mock injection of 0.48 µL followed by 16 injections of 2.97 µL, with 250 s spacing and a 260 rpm stir rate. A blank was measured by repeating the titration scheme using 20 mM sodium phosphate, pH 7.4 alone both in the syringe and in the cell. The NanoAnalyze software (TA instruments) was used to subtract the baseline from the raw data and analyze the binding isotherm using the independent model, where the reaction stoichiometry (n parameter) was fixed at a value of 1 (differential enthalpy estimation inaccurate due to c parameter ≃ 0.3).

Structure of (P3-7)$_n$-like motifs in AlphaFold and ColabFold

The UniprotKB accession codes of proteins containing (P3-7)n-like motifs identified using ScanProsite (see methods) were used to retrieve structural models from the AlphaFold Database[6], in which the secondary structure of the (P3-7)n-like motifs fulfilling our design rules was obtained using DSSP[7]. In addition, we predicted the structure of a subset of 42 of these proteins using ColabFold[8] 1.3.0 with and without multiple sequence alignment[11] and using the AlphaFold2 ptm model[12] (Supplementary Figure 7d). The models were relaxed with a short amber molecular dynamics trajectory (using `--amber` in ColabFold).

Supplementary References

1. Wei, X. *et al.* Retro-inverso isomer of Angiopep-2: a stable d-peptide ligand inspires brain-targeted drug delivery. *Mol. Pharm.* **11**, 3261–3268 (2014).
2. Liu, A. *et al.* Hydrogen-bond detection, configuration assignment and rotamer correction of side-chain amides in large proteins by NMR spectroscopy through protium/deuterium isotope effects. *Chembiochem* **9**, 2860–2871 (2008).
3. Li, D.-W. & Brüschweiler, R. PPM: a side-chain and backbone chemical shift predictor for the assessment of protein conformational ensembles. *J. Biomol. NMR* **54**, 257–265 (2012).
4. de Castro, E. *et al.* ScanProsite: detection of PROSITE signature matches and ProRule-associated functional and structural residues in proteins. *Nucleic Acids Res.* **34**, W362–5 (2006).
5. UniProt Consortium. UniProt: the universal protein knowledgebase in 2021. *Nucleic Acids Res.* **49**, D480–D489 (2021).
6. Tunyasuvunakool, K. *et al.* Highly accurate protein structure prediction for the human proteome. *Nature* **596**, 590–596 (2021).
7. Kabsch, W. & Sander, C. Dictionary of protein secondary structure: pattern recognition of hydrogen-bonded and geometrical features. *Biopolymers* **22**, 2577–2637 (1983).
8. Mirdita, M. *et al.* ColabFold: making protein folding accessible to all. *Nat. Methods* **19**, 679–682 (2022).
9. Chen, V. B. *et al.* MolProbity: all-atom structure validation for macromolecular crystallography. *Acta Crystallogr. D Biol. Crystallogr.* **66**, 12–21 (2010).
10. Escobedo, A. *et al.* Side chain to main chain hydrogen bonds stabilize a polyglutamine helix in a transcription factor. *Nat. Commun.* **10**, 2034 (2019).
11. Mirdita, M., Steinegger, M. & Söding, J. MMseqs2 desktop and local web server app for fast, interactive sequence searches. *Bioinformatics* **35**, 2856–2858 (2019).
12. Jumper, J. *et al.* Highly accurate protein structure prediction with AlphaFold. *Nature* **596**,

583–589 (2021).
