# Supplementary material for: A glutamine-based single α-helix scaffold to target globular proteins
Source: Nat Commun. 2022 Nov 18;13:7073. doi: 10.1038/s41467-022-34793-6 (PMC9674830; doi:10.1038/s41467-022-34793-6)
Supplement: Supplementary file 3 — Description of Additional Supplementary Files [file 41467_2022_34793_MOESM3_ESM.pdf]

Supplementary Data 1. Solution structure of peptide (P3-7)$_2$. Probability densities for side chain χ_1 and χ_2 torsion angles were derived from a per-residue analysis of a 3D CNH-NOESY spectrum using CoMAND and two MD-derived frame pools and used to train a Gaussian mixture model (GMM). A structural ensemble of peptide (P3-7)$_2$ was then built through CoMAND R-factor minimization by selecting backbone conformations from both frame pools along with side chain conformations generated by GMM sampling. This is one out of 20 ensembles generated, shown in Figure 2g and available as PDB entry 8B1X.

Supplementary Data 2. All generated structural ensembles of peptide (P3-7)$_2$. All structural ensembles were generated to an equivalent R-factor minimization and used for RDC back-calculation.

Supplementary Data 3. Dataset of (P3-7)$_n$-like motifs. ScanProsite was used to query UniProtKB for the presence of (P3-7)$_n$-like motifs in natural sequences. Here we enclose a list of all 3451 identified motifs along with their UniProt accession codes and taxonomic details. Details are also provided on the 2303 of these sequences for which we found structural models in the AlphaFold Database, as well as the 42-protein subset we calculated structural models for with and without MSAs using ColabFold.

Supplementary Data 4. Structural models of proteins containing (P3-7)$_n$-like motifs calculated with and without MSAs using ColabFold. Structural models for a subset of 42 proteins bearing (P3-7)$_n$-like motifs were calculated with and without MSAs using ColabFold, showing that the helicity of such motifs is encoded in AlphaFold learnt structural preferences.
